# Comprehensive genome-wide analysis of the *HMGR* gene family of *Asparagus taliensis* and functional validation of *AtaHMGR10* under different abiotic stresses

**DOI:** 10.3389/fpls.2025.1455592

**Published:** 2025-02-20

**Authors:** Liangqin Zeng, Sylvia E. Brown, He Wu, Wenhua Dongchen, Yunbin Li, Chun Lin, Zhengjie Liu, Zichao Mao

**Affiliations:** ^1^ College of Agronomy and Biotechnology, Yunnan Agricultural University, Kunming, China; ^2^ College of Metallurgical and Chemical Engineering, Kunming Vocational and Technical College of Industry, Kunming, China; ^3^ Institute of Improvement and Utilization of Characteristic Resource Plants, Yunnan Agricultural University, Kunming, China; ^4^ The Laboratory for Crop Production and Intelligent Agriculture of Yunnan Province, Yunnan Agricultural University, Kunming, China

**Keywords:** *Asparagus taliensis*, *HMGR* gene family, transgene, abiotic stress tolerance, medicinal plant

## Abstract

**Introduction:**

Hydroxy-3-methylglutaryl-coenzyme A reductase (HMGR) is a key enzyme in the terpenoid biosynthetic pathway, playing a crucial role in plant stress responses. However, the *HMGR* gene family in *Asparagus taliensis*, a traditional Chinese medicinal herb with high steroidal saponin content and strong stress tolerance, remains poorly understood. This study investigates the stress response mechanisms of the *HMGR* gene family in *A. taliensis* under abiotic stress conditions.

**Methods:**

A comprehensive genome-wide analysis of the *HMGR* gene family in *A. taliensis* was conducted. The analysis included chromosomal localization, phylogenetic tree construction, linear analysis, gene structure characterization, motif distribution, cis-acting elements, and protein structure. Candidate *AtaHMGR10* gene were overexpressed in *Arabidopsis thaliana* to analyze phenotypic changes under osmotic and salt stress, including seed germination rate and primary root length. Physiological parameters were also analyzed, and gene expression was validated using qPCR under drought, osmotic, and salt stress conditions.

**Results:**

A total of 18 *HMGR* gene family members were identified in *A. taliensis*. The functions and evolution of *AtaHMGR* genes are conserved. *AtaHMGR10* was selected as a promising candidate due to its unique expression profile. Docking analysis revealed that *AtaHMGR10* has conserved motifs for binding both HMG-CoA and NADPH/NADH, showing equal affinity for both. Overexpression of *AtaHMGR10* in transgenic *A. thaliana* enhanced tolerance to abiotic stresses, as evidenced by higher germination rates, improved primary root length, increased chlorophyll and proline levels, enhanced peroxidase (POD) and catalase (CAT) activities, and reduced malondialdehyde (MDA) content compared to non-transgenic plants under stress conditions.

**Discussion:**

These findings highlight the role of *AtaHMGR10* in enhancing plant stress tolerance, particularly in combating drought, osmotic, and salt stress. This understanding of its potential function provides avenues for improving crop resilience to abiotic stress through future gene modification.

## 1 Introduction

Abiotic stresses such as drought, cold and high salinity impact growth, development as well as distribution of plants. To counter these challenges, plants have developed a variety of adaptive mechanisms at the molecular, cellular, physiological, and biochemical levels. Among these, terpenoids, especially steroidal saponins are key secondary metabolites that have critical roles against stresses. In Angiosperm plants, isopentenyl diphosphate (IPP) and dimethylallyl diphosphate (DMAPP) as basic five carbon unit for biosynthesis of terpenoids (e.g. steroidal compounds and phytohormones) are synthesized by two different pathways: the mevalonate (MVA) pathway in the cytoplasm and the 2-C-methyl-D-erythritol-4-phosphate pathway (MEP) in plastids (Kuravadi et al., 2015; Pandreka et al., 2015). HMGR, which is a rate-limiting enzyme in the MVA pathway (Segatto et al., 2014), catalyzes the conversion of 3-hydroxy-3-methylglutaryl-CoA (HMG-CoA) to MVA using two molecules of nicotinamide adenine dinucleotide phosphate (NADPH) or nicotinamide adenine dinucleotide (NADH) as reducing agents (Bach, 1986; Segatto et al., 2014). The MVA pathway is responsible for producing terpenoids e.g., artemisinin-a natural antimalarial drug isolated from the Chinese medicinal plant *Artemisia annua* (Li et al., 2023).

Recent research have underscored the pivotal functions of the HMGR in catalyzing terpenoids synthesis across a variety of plant species. In *Azadirachta indica*, overexpression of *AiHMGR2* directly correlated with the production of azadirachtin, a terpenoid with notable pesticidal properties (Bhambhani et al., 2017). Overexpression of *AaHMGR* in *Artemisia annua* resulted in increased artemisinin content (Li et al., 2024), while overexpression of *OkHMGR* in *Ocimum kilimandscharicum* enhanced the production of aromatic terpenoids (Bansal et al., 2018). Similarly, in hybrid *Populus*, elevated expression of *PtHMGR* led to increased terpenoid levels, enhancing the plant’s resistance to stress (Wei et al., 2019). These findings demonstrate the roles of *HMGR* genes in the biosynthesis of terpenoids and isoprenoids for plants’ adaptation to diverse environmental signals.

Environmental stresses and developmental signals (e.g., phytohormones) profoundly affect plant physiology. *HMGR* genes are normally upregulated under such conditions, enhancing stress-related biosynthesis of metabolites. For example, *SmHMGR2* and *SmHMGR3* were positively regulated by phytohormonal stimulations (Ma et al., 2012). *MdHMGR4* demonstrated a root-specific expression pattern in apple and its expression was significantly enhanced by phytohormonal treatments (Lv and Zhang, 2017). In rice, drought treatment upregulated *OsHMGR* genes, facilitating phytosterol synthesis, thereby enhancing the plant’s resistance to drought conditions (Kumar et al., 2015, 2018). Overexpression of *MdHMGR5* in apple increased plant tolerance to oxidative stress by enhancing reactive oxygen species (ROSs) scavenging abilities in transgenic plants (Zhang et al., 2020). Similarly, overexpression of *PtHMGR* in *Populus* led to remarkable decrease in membrane damage under drought stress (Wei et al., 2020). Moreover, the expression of *HMGRs* were positively regulated by hormonal treatment, as demonstrated in grapes where *VvHMGR1* maintained high expression levels potentially linked to growth and stress-related phytohormones synthesis and signaling (Zheng et al., 2021). Additionally, *PtHMGR5* is reported to be involved in the increased tolerance to drought in *Populus* (Ma et al., 2023). β-Sitosterol, its precursors of biosynthesis was catalyzed by *EkHMGR*, was reported to enhance plant drought tolerance (Wang et al., 2024), therefore *HMGRs* have been applied to enhance plant stress tolerance by transgenic approaches. Previous studies have shown that overexpression of *HMGR* enhances resistance to abiotic stresses in various plant species. However, little is known about *Asparagus taliensis*, a species of the genus *Asparagus* (Asparagaceae) endemic to Yunnan, China, which has traditionally been used as herbal medicine due to the presence of steroidal saponins, which have anti-inflammatory properties, ability to induce cancer cells apoptosis and modulation of immune responses (Podolak et al., 2010; Sparg et al., 2004).

*Asparagus* L. contains more than 200 species distributed across the old world, with southern Africa being the cradle center and southwestern China, regarded as the diversity center of dioecious *Asparagus* species (Wu et al., 2024) among which 8 dioecious species including, *A. taliensis* are endemic in Yunnan province of China (Saha et al., 2015). The phylogenetic study showed that in the dioecious *Asparagus* species of Eurasia, *A. taliensis* clustered with *Asparagus conchinchinensis* into a small clade, suggesting a closer phylogenic relationship (Bentz et al., 2024a). *A. taliensis* exhibits strong tolerance to environmental stresses, such as drought and cold, as it grows well in the low precipitation winters of Yunnan. The tuber roots of *A. taliensis*, which are used for medicinal purposes, are larger than those of *A. cochinchinensis*, making it an alternative plant for *A*. *cochinchinensis* replacement planted in wide regions of China for higher yields. It is also regarded as an ideal germplasm for breeding of *A. cochinchinensis* through interspecific hybridization due to their closer phylogenetic relationship. Based on our assembled and annotated *A. taliensis* genome (unpublished), this study involved a comprehensive analysis of the *HMGR* gene family in *A. taliensis*, and the *AtaHMGR10* was predicted as a candidate of significant interest due to its unique expression profile, the potential function inferred from its conserved motifs for the binding HMG-CoA and NADPH/NADH, as well as the equivalent binding abilities of both NADPH and NADH by Alphafold3 docking analysis respectively. Furthermore, the overexpression of *AtaHMGR10* in transgenic *A. thaliana* significantly improved tolerance to both drought and salt treatments. Overall, our research provides critical insights to the *HMGR* gene family in *A. taliensis*, contributing to the understanding of its evolutionary and functional roles in enhancing abiotic stress tolerances. This study also offers a valuable gene source for improving germplasm in *Asparagus* species, promoting the biosynthesis of medicinal terpenoids in microbial hosts using synthetic biology methods, and enhancing stress tolerance in crops through genetic modification.

## 2 Materials and methods

### 2.1 Plant materials

The seeds of *A. taliensis* were collected from a cultivation field in Luxi County, Yunnan, China, and the germinated seedlings were transplanted in the field of Yunnan Agricultural University. The 4-month-old seedlings, were treated with 200 μmol/ml jasmonic acid (MeJA) for 72 hrs. Non-MeJA-treated seedlings (control) and MeJA-treated seedlings (12 hr, 24 hr, 36 hr, 48 hr, 60 hr, and 72 hr), along with fresh roots (Rs), stems (Ss) and flowering twigs (Fs) from two-year-old field plants, were sampled with three biological replicates for each. The samples were immediately frozen in liquid nitrogen and stored at -80°C for subsequent RNA extraction and RNA sequencing analysis, as reported in our previous study (Wu et al., 2024).

*A. thaliana* was used as the transgenic host (wildtype, WT) and the *AtaHMGR10* transgenic Arabidopsis plants were generated with the floral dip method (Zhang et al., 2006). The transgenic and WT *A. thaliana* seeds were surface-sterilized with 75% (v/v) ethanol for 1 minute, rinsed five times with sterile water, and sown on 1/2 Murashige and Skoog (MS) medium (Solarbio, Beijing, China) (pH 5.8). After undergoing vernalization for two days at 4°C, seeds were cultured for seedling preparation in a controlled environment chamber under a 14-hour light at 24°C/10-hour dark at 22°C, maintaining a relative humidity of 60%-70%. The seedlings were transplanted into pods and grown under the same germination conditions as above. Six-week-old WT and transgenic *A. thaliana* lines were selected and exposed to drought, salt, and osmotic stresses respectively. Plant samples were collected, frozen in liquid nitrogen, and stored at -80°C or directly used for phenotypical observations, as well as physiological, biochemical, and molecular detections.

### 2.2 Genome-wide identification of *HMGR* genes in *A. taliensis*

Based on our assembled and annotated genome of *A. taliensis* (unpublished), the *HMGR* family members were predicted through hmmsearch with HMMER (v3.0) software (Finn et al., 2011) using the HMGR Hidden Markov Model (PF00368), downloaded from the Pfam database (Blum et al., 2021) as well as the homologous search by BLASTP with BLAST (v2.11.0+), using query of 12 function confirmed *HMGR* genes from various plants (e.g. *A. thaliana and Oryza sativa*), with filtering critical standard set as: similarity > 40%, coverage > 80% and e-values <1e^-6^ (McGinnis and Madden, 2004). The conserved domains in the resulted HMGR proteins were further confirmed through comparison with the database of InterPro (Lu et al., 2020). The physicochemical properties of the predicted *AtaHMGR* members were analyzed using TBtools software (v2.142) (Chen et al., 2023). Subcellular localization of HMGR proteins was predicted using the web tools of WoLF PSORT (Horton et al., 2007).

### 2.3 Analysis of chromosome localization and synteny of *AtaHMGRs*

Chromosomal localization of the *AtaHMGR* genes was performed using the MapGene2Chrom (MG2C) tool (http://mg2c.iask.in/mg2c_v2.1/). The calculation of non-synonymous (Ka) and synonymous (Ks) substitutions, along with their ratios (Ka/Ks), were computed using kakscalculator2 (https://github.com/kullrich/kakscalculator2). Additionally, synteny analysis for the *AtaHMGR* family members were conducted with the MCScanX tool (Wang et al., 2013), and outputs were visualized with Circos software (https://github.com/node/circos).

### 2.4 Phylogenetic analysis of HMGR proteins

Genomic sequences of *A. thaliana* (GCA_028009825.2), *Nymphaea colorata* (GCF_008831285.2), *Dioscorea zingiberensis* (GCA_026586065.1), *Asparagus racemosus (GCA_037177215.1)*, *Asparagus setaceus (GCA_012295165.1)*, and *Asparagus officinalis (GCF_001876935.1)* were retrieved from the NCBI genome database. Multiple sequence alignments of candidate HMGR proteins were performed using the MUSCLE algorithm with default parameters (Edgar, 2004). A phylogenetic tree was then constructed with MEGA-X software (Kumar et al., 2018b), using the maximum likelihood method with 1000 bootstrap replicates to estimate evolutionary distances. The resulting phylogenetic tree was visualized and refined using the iTOL online tool (Letunic and Bork, 2021).

### 2.5 Gene structure, conserved motif and cis-acting element analysis of *AtaHMGRs*

Conserved motifs were analyzed employing the Multiple Expectation Maximization for Motif Elicitation (MEME Suite) (Bailey et al., 2015). The maximum number of motifs considered was limited to 20 motifs, width of motifs: 6-100, with default parameters. Information on the exon-intron structure for *AtaHMGRs* was derived from genomic annotated GFF files as well as the manual corrections by BAM file resulted from RNAseq mapping by Hisat2 (Thakur, 2024). Cis-elements of promoter, extending 2000 bp upstream from the start codon (ATG), were predicted using the PlantCARE database (Lescot et al., 2002).

### 2.6 Analysis folding structures, substrates docking of *AtaHMGRs*

The secondary structure of proteins was predicted using the online tool SOPMA (Geourjon and Deleage, 1995). Folding of the 3D structures for *AtaHMGRs* as well as their substrate binding sites were conducted using the Alphafold3 (https://alphafoldserver.com/) (Callaway, 2024; Abramson et al., 2024) or SWISS-MODEL (https://swissmodel.expasy.org/), and the resulting 3D structure of 18 *AtaHMGRs* were visualized using PyMOL (Schrodinger, 2015).

### 2.7 Expression profiles analysis of *AtaHMGRs*

The expression patterns of *AtaHMGRs* were assessed in three distinct tissues (Fs, Rs, and Ss) of two-year-old field-grown *A. taliensis*, as well as in four-month-old seedlings grown in pots with or without MeJA treatments. The MeJA-treated seedlings were compared to untreated controls at seven different time points (0, 12, 24, 36, 48, 60, and 72 hours). The total RNAs were extracted by RNAPrep pure plant kit (TianGen, Beijing, China), then reverse transcribed to cDNA using GoScript™ Reverse Transcription Mix, Random Primer kit (Promega, Beijing China) according to the manufacturer’s protocols. The cDNA (~10 μg) was sequenced using the Illumina Genome Analyzer II platform (Illumina, San Diego, USA) with libraries of 300 bp cDNA insertions with 150 bp pair-end (PE) model. All resulted RNAseq data were evaluated with FastQC (https://github.com/topics/fastqc), then filtered with Fastp v0.23.4 (https://github.com/OpenGene/fastp) to get clean data. The clean reads were mapped to the genome of *A. taliensis* using hisat2 (v2.2.1) (Kim et al., 2015) resulting in BAM files. Expression quantification was performed through scripts of FeatureCounts (Liao et al., 2014) to obtain matrix of TPM (standardized expression units of transcripts per kilobase of exon model per million mapped reads). The expression profiles were visualized using the TBtools software.

### 2.8 Cloning, vector construction and Arabidopsis transformation of *AtaHMGR10*

Total RNA of *A. taliensis* was isolated with the RNA Easy Fast Plant Tissue Kit, and the quality and concentration were assessed using the KRIRO K5600 (Kaiao Biotech, Beijing, China). cDNA was obtained using the FastKing gDNA dispelling RT SuperMix. The full coding sequence (CDS) of *AtaHMGR10* was amplified with the primers *AtaHMGR10*-1F and *AtaHMGR10*-1R and cloned into T-vector. After the sequence correction was confirmed by Sanger sequencing, the CDS of *AtaHMGR10*, was reamplified with primers *AtaHMGR10*-2F and *AtaHMGR10*-2R, and cloned into the pBWA(V)HS vector to obtain expression vector with *AtaHMGR10* controlled by a CaMV 35S promoter. The constructed expression vector, was introduced into *Agrobacterium tumefaciens* GV3101, then subsequently transformed into *A. thaliana* Col-0 plants *via* the floral dip method (Narusaka et al., 2012). Screening for hygromycin resistance (20 mg/L) was conducted to select hygromycin resistant plants, and transgenic Arabidopsis plants were further confirmed by PCR amplification with primers *AtaHMGR10*-1F and *AtaHMGR10*-1R. All primers used in this study are listed in **Supplementary Table S1**, and the transgenic Arabidopsis plants were cultivated until second generation of transgenic plant (T2) seeds and harvested for further phenotypical observations.

### 2.9 Subcellular localization of *AtaHMGR10* in tobacco protoplasts

For detection of *AtaHMGR10* subcellular location, pBWA(V)HS -GFP vector was used to construct a fused *AtaHMGR10*-GFP expression vector. In detail, the coding region of *AtaHMGR10* (excluding the stop codon) was amplified by PCR from cDNA with primers of *AtaHMGR10*-3F and *AtaHMGR10*-3R (listed in **Supplementary Table S1**), cloned into the pBWA(V)HS-GFP vector with double digestion of both *BgI* II and *Spe* I, resulting in pBWA(V)HS-*AtaHMGR10*-GFP vector. Protoplasts were isolated from *Nicotiana tabacum* leaves and underwent polyethylene glycol (PEG)-mediated transformation (Antolín-Llovera et al., 2011). The procedure began by carefully peeling the upper epidermal layer of the leaves and immersing it in an enzymatic solution containing 1.5% cellulase R10, 0.4% macerozyme R10, 0.4 M mannitol, 20 mM KCl, and 10 mM MES-KOH (pH 5.8), which was gently agitated for 10 minutes in the dark. Subsequently, the mixture was filtered through a nylon mesh and washed twice using W5 solution (154 mM NaCl, 125 mM CaCl_2_, 2 mM KH_2_PO_4_, 2 mM MES, 5 mM glucose, pH 5.7-5.8). The protoplasts were then resuspended in MMG solution (0.4 M mannitol, 15 mM MgCl_2_·6H_2_O, 4 mM MES-KOH, pH 5.7-5.8) to achieve a density of ~ 2×10^5^ cells/mL. For the transformation process, 10 µg of plasmid DNAs was mixed with 100 µL of the protoplast suspension, followed by adding 110 µL of PEG solution (40% PEG4000, 0.2 M mannitol, 100 mM CaCl_2_). This mixture was incubated at room temperature for 10-15 minutes in the dark, and then diluted with 1 mL of W5 solution. After centrifugation at 300 rpm for 3 minutes, the collected pellets were washed once or twice with 1 mL of W5 solution, resuspended in the same 1 mL W5 solution, and incubated at 25°C for 18-24 hours. GFP fluorescence signals were observed and images were captured using a confocal scanning microscope (C2-ER, Nikon, Japan).

### 2.10 Drought, osmotic, salt stress treatments and statistical analysis

*AtaHMGR10* transgenic *A. thaliana* seeds (lines OE-1 and OE-3 of T2 generation) were utilized to assess the germination rates under salt and osmotic stress. All experiments were performed in triplicate. Both WT and transgenic *A. thaliana* seeds were surface-sterilized and plated on 1/2 MS medium supplemented with 0 & 50 mM NaCl, and 0 & 200 mM mannitol, respectively. The plates were then incubated in a growth chamber, and germination rates were determined after 9 days.

The plants of WT and transgenic *A. thaliana*, aged six weeks, were exposed to drought for 0, 5, and 10 days, with subsequent re-watering for 6 days. Leaves were sampled to assess drought impacts. To gauge the salt and osmotic stress responses, leaves were also sampled at 0, 3, 6, 12, 24, and 36 hours following exposure to 50 mM NaCl and 10% PEG 6000 treatments respectively. These intervals were selected to monitor the immediate stress response dynamics. Biochemical analyses of total chlorophyll, MDA, proline, and POD & CAT activities were conducted using a Boxbio reagent kit (Boxbio Science & Technology, Beijing, China), following the protocols of the manufacturer.

Data collection was facilitated using Microsoft Excel, and statistical analysis was conducted using two-way ANOVA through GraphPad Prism version 10.1.2. This analysis method is consistent with standard practices for evaluating interactive effects in biological experiments. The results were presented as mean ± standard deviation (SD) from three biological replicates. Statistical significance was defined as *P < 0.05 and **P < 0.01 when compared to the control.

### 2.11 Quantitative real-time PCR analysis

The extracted total RNA was reverse transcribed into cDNA using Hifair^®^ II 1st Strand cDNA Synthesis Kit (11119ES60, Yeasen, China). qRT-PCR assays were conducted using ChamQ Universal SYBR qPCR Master Mix(Q711, Vazyme, China) with a QuantStudio 5 Real-Time PCR System (Applied Biosystems). A 20 μL PCR system includes: 10.0 μL 2×ChamQ Universal SYBR qPCR Master Mix, 0.4 μL for each primer (10 μM), 2 μL templates cDNA and 7.2 μL H_2_O. PCR thermocycle parameters were performed as following: 95°C for 30 s; 40 cycles of 95°C for 10 s, 60°C for 30 s, and 95°C for 15 s, 60°C for 60 s, 95°C for 15 s. The assay was set up with three biological replicates each, employing the 2^−ΔΔCt^ method to calculate relative expression levels. The primers for qRT-PCR detections were listed in **Supplementary Table S1**.

## 3 Results

### 3.1 Genome-wide identification and characterization of *AtaHMGR* genes in *A. taliensis*

18 members of *HMGR* gene family, designated *AtaHMGR1*–*AtaHMGR18* including their chromosomal position, were identified in *A. taliensis*, and these identified family members were further analyzed for their amino acid (AA) composition, molecular weight (MW), theoretical isoelectric point (pI), aliphatic index, grand average of hydropathicity (GRAVY), instability index, subcellular localization, and other physiochemical characteristics (**Supplementary Table S2**). The results showed that the gene lengths among the 18 *AtaHMGRs* ranged from 516 bp (*AtaHMGR6*) to 7,017 bp (*AtaHMGR10*), and coding DNA sequences (CDSs) ranged from 276 bp (*AtaHMGR2*) to 1,722 bp (*AtaHMGR8*). Protein lengths ranged from 91 AAs (*AtaHMGR2*) to 573 AAs (*AtaHMGR8*) with averaging 280 AAs. Four proteins (*AtaHMGR8*, *AtaHMGR10*, *AtaHMGR11*, and *AtaHMGR18*) exceeded 500 AAs. Molecular weights ranged from 9,653.31 Da (*AtaHMGR2*) to 61,010.1 Da (*AtaHMGR10* and *AtaHMGR11*), with those of *AtaHMGR8*, *AtaHMGR10*, *AtaHMGR11*, and *AtaHMGR18* surpassing 50 kDa, indicating potential basic catalytic roles in MVA pathway with intricate cellular functions. The PI values ranged from 5.14 (*AtaHMGR9*) to 9.85 (*AtaHMGR12*), with most above 7, hinting at a predominance of basic amino acids compositions in this family. The instability index varied from 38.1 (*AtaHMGR15*) to 66.4 (*AtaHMGR12*) with proteins under 40 considered stable, while GRAVY values varied from -0.181 (*AtaHMGR6*) to 0.659 (*AtaHMGR14*), and the aliphatic index showed values between 79.77 (*AtaHMGR7*) and 117.73 (*AtaHMGR14*), with 11 proteins having an index above 90, indicative of high diversities of thermal stability as well as membrane binding abilities of *AtaHMGRs*. Predictive subcellular localization analyses from WoLF PSORT web showed that 5/18 *AtaHMGRs* were localized to the plasma membrane, 4/18 in the vascular membrane, 3/18 in the cytosol and 2/18 were located in the chloroplast (CP), endoplasmic reticulum (ER) and mitochondrion (MT) respectively suggesting the expression, location and functional diversity of *AtaHMGRs*.

### 3.2 Chromosomal location and synteny analyses of *AtaHMGRs*

The resulting chromosomal locations of *HMGRs* in *A. taliensis* showed that the 18 *AtaHMGR* genes were distributed across 8 chromosomes (Chrs) (**Supplementary Figures S1**, **S2A**). Chr05 harbors 5 genes of *AtaHMGRs*, accounting for 27.78%, while Chr 01, 03, and 08 each has 2 genes accounting for 33.3%, in contrast, Chr 02, 04, 06 and 07 has only a single gene each (22%). No *AtaHMGR* genes were located on Chr 09 and 10, while three of these genes (*AtaHMGR16*, *AtaHMGR17* and *AtaHMGR18*, accounting for 16.6%) were located on contigs which were not anchored into Chrs (**Supplementary Figure S1**).

To elucidate the possible phylogenetic relationships and evolutionary dynamics across angiosperm species, six representative species, includes one basal angiosperm species, *N. colorata*, one representative dicot species, *A. thaliana*, and four saponin-producing monocot species (i.e., *D. zingiberensis*, *A. setaceus*, *A. racemosus* and *A. officinalis*) were selected for collinear analysis alongside *A. taliensis.* The results showed that *A. taliensis* shares only one collinear gene pair each with *N. colorata* and *A. thaliana*; however, multiple collinear gene pairs were found in steroidal saponins producing species having five orthologous gene pairs with *D. zingiberensis*, *A. racemosus* and *A. officinalis* respectively, but had four orthologous gene pairs with *A. setaceus* (**Supplementary Figure S2B**, **Supplementary Table S3**). Interestingly, *A. taliensis* exhibits an elevated number of synteny connections with saponins producing species indicting *AtaHMGRs* play roles in steroidal saponins biosynthesis and accumulation. *A. taliensis* maintains certain levels of collinearity with *N. colorata* and *A. thaliana* (no steroidal saponins production), possibly reflecting older divergence events before the monocot evolution. The observed synteny among such divergent angiosperm taxa suggests that some *HMGR* gene family members are highly conserved across extensive evolutionary events, fulfilling pivotal roles in terpenoids (e.g., cholesterol, brassinosteroids (BRs) and volatile terpenes) metabolism that have persisted through the course of angiosperm evolution. This study also showed that the orthologous gene pairs of *AtaHMGR* to selected species have either a one-to-many or many-to-one homology models, specifically, three *AtaHMGR* genes (*AtaHMGR8*, *AtaHMGR10*, and *AtaHMGR11*) displayed collinear associations with more orthologous gene pairs in steroidal saponins producing monocot species (**Supplementary Tables S3**, **S4**). However, the study showed no collinear relations for the remained 15 *AtaHMGR* genes, suggesting that these genes may have been newly duplicated after the species generation of *A. taliensis*. Based on the multiple gene duplication events observed in current study also suggest the closer phylogenetic ties among the examined saponins producing species in this study.

Gene repetition events in *A. taliensis* were further analyzed using the MCScanX tools (Wang et al., 2012). The results revealed 40 gene pairs exhibiting gene duplication during the evolutionary history (**Supplementary Figure S2A**, **Supplementary Table S4**). Eight genes (*AtaHMGR1*, *AtaHMGR5*, *AtaHMGR7*, *AtaHMGR8*, *AtaHMGR10*, *AtaHMGR12*, *AtaHMGR16*, and *AtaHMGR17*) arose from WGD/segmental duplications. The *AtaHMGR11* may have resulted from proximal segmental duplication, typically involving gene duplication in neighboring chromosomal regions. In contrast, nine genes (*AtaHMGR2*, *AtaHMGR3*, *AtaHMGR4*, *AtaHMGR6*, *AtaHMGR9*, *AtaHMGR13*, *AtaHMGR14*, *AtaHMGR15*, and *AtaHMGR18*) underwent dispersed duplication model. The analysis of the nonsynonymous-to-synonymous substitution rate ratio (Ka/Ks) provided insights into the evolutionary dynamics and selective pressures affecting these genes. The Ka/Ks ratios, calculated by *A. taliensis* comparison to the *HMGR* orthologs in six selected species (**Supplementary Table S4**) were below 1, consistent with purifying selection and indicative of the evolutionary preservation of these genes.

In summary, these HMGR family member genes likely retain conservative functions across angiosperm taxa indicating that these genes keep the same ancestral roles of terpenoids synthesis even throughout long historical replication, following variations, truncked coding and even removal for environmental adaptation.

### 3.3 Phylogenetic analysis of *HMGR* family genes

**Figure 1:**
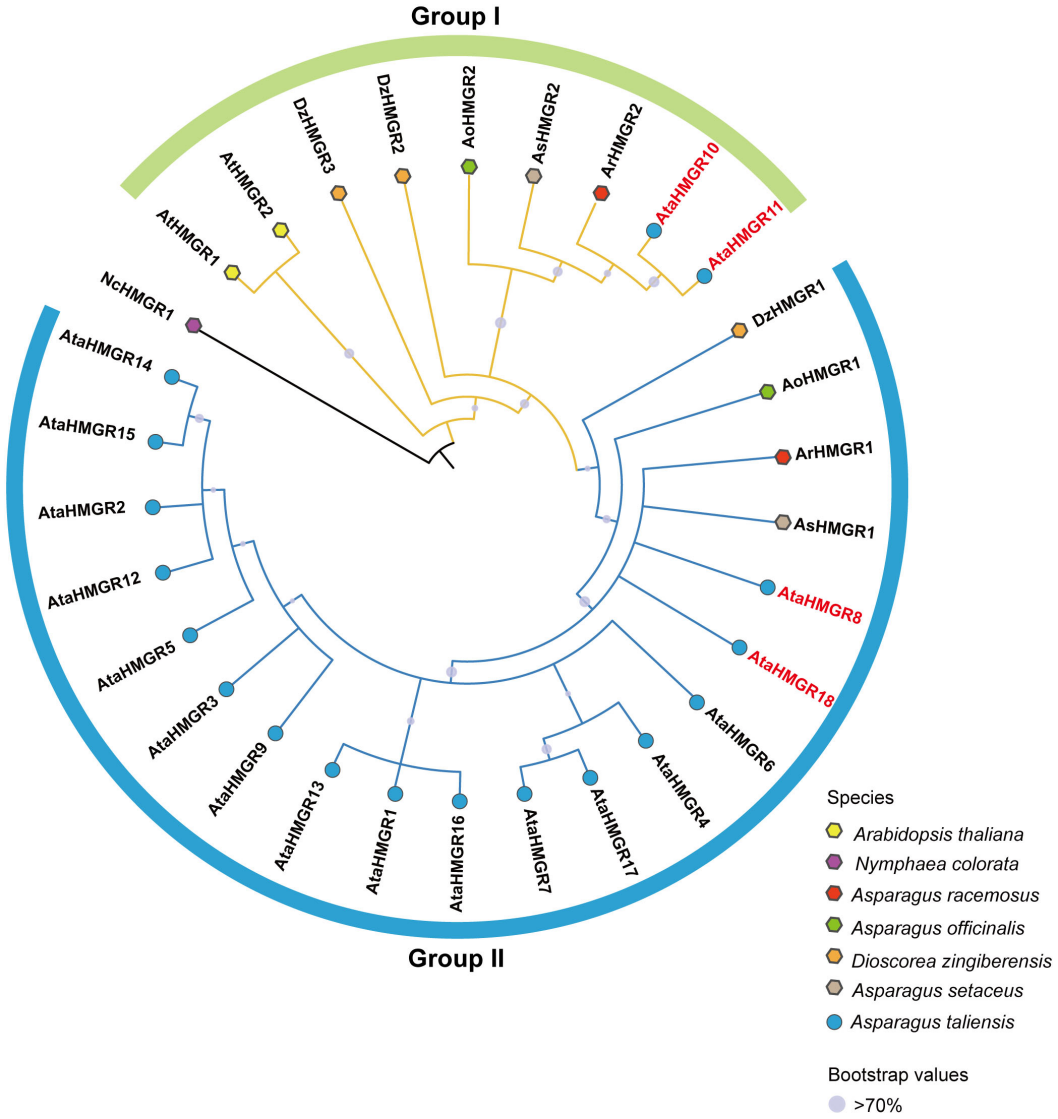
Phylogenetic relationship of HMGR proteins among selected plant species including: *A. taliensis*, *A. thaliana*, *N. colorata*, *A. racemosus*, *A. officinalis*, *D. zingiberensis*, and *A. setaceus* constructed with MEGA-X. Bootstrap confidence values are symbolized by dark dots, with values of 70 or above indicating substantial statistical support. The size of each circle is directly proportional to its bootstrap value, reflecting the relative confidence in the phylogenetic inference of each clade.

To reveal the evolutionary relationships of the HMGR proteins, a total of 30 HMGR protein sequences were used to construct a phylogenetic tree (**Supplementary Table S5**). The phylogenetic tree, rooted with *NcHMGR1* from N.colorata, categorized the *HMGR* gene family into two major groups: Group I and Group II (**Figure 1**).Within Group I, *A. taliensis HMGR* members, *AtaHMGR10* and *AtaHMGR11*, formed a distinct cluster, whereas the remaining 16 *AtaHMGR* genes were grouped in Group II. This distribution suggests that the *HMGR* gene family in *A. taliensis* may have undergone divergent evolutionary pathways, with *AtaHMGR10* and *AtaHMGR11* potentially representing a lineage distinct from the other genes. Furthermore, *HMGR* genes from steroidal saponins producing species (*A. officinalis*, *A. setaceus*, *A. racemosus* and *D. zingiberensis*) were distributed across both Group I and Group II. This cross-group pattern indicates that *HMGR* genes in these species may have experienced multiple independent evolutionary events or been influenced by varying selective pressures, contributing to the phylogenetic diversity observed within the gene family.

### 3.4 Conserved motifs analysis of *AtaHMGRs*

**Figure 2:**
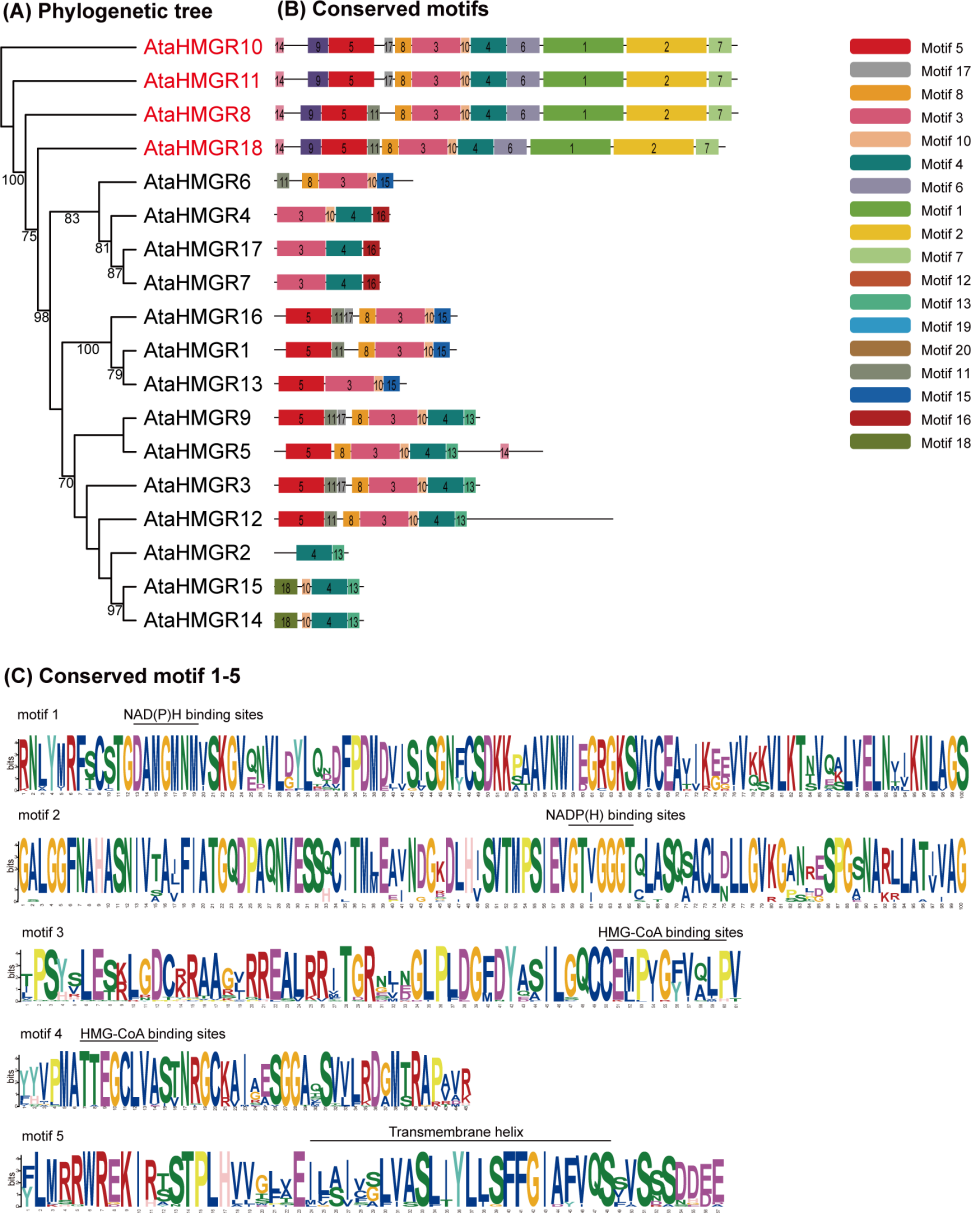
Phylogenetic and conserved motif analysis of *HMGR* members. **(A)** The phylogenetic tree of the 18 *AtaHMGRs*; **(B)** The distribution of the conserved motifs indicated by different colors; **(C)** Five conserved motif logos including a transmembrane helix, HMG-CoA binding sites and NAD(P)H binding sites.

The present study delineates the structural variations of the *HMGR* gene family members in *A. taliensis*, based on the comprehensive evaluation of conserved motifs. A significant phylogenetic relationship among the 18 genes of the *AtaHMGR* family were discerned (**Figure 2A**). A total of 20 conserved motifs, labeled motifs 1–20 (**Figure 2B**) were identified, which are variably present across these gene coding proteins. The results also showed that 5 conserved motifs are predominantly observed: motif 1 annotated to a NAD(H)/NADP(H), named as NAD(P)H, binding site (with conserved motif DAMGMNM, same as below), motif 2 to another NAD(P)H binding site (GTVGGGT), motif 3 to a HMG-CoA binding site (EMPVGFVQLP), motif 4 to another HMG-CoA binding site (TTEGCLVA) and motif 5 to a transmembrane helix (ILAIVSLVASLIYLLSFFGIAFVQS) (**Figure 2C**). Interestingly, the results also showed that the motif 3 (EMPVGFVQI) differs with an individual amino acid residue where tyrosine (Y) was replaced by phenylalanine (F) in comparison with the previously reported sequence (EMPVGYVQI) (Liu et al., 2018). This variation is within the permissible range for the binding ability of HMG-CoA in *AtaHMGR* functionality, considering the similar biochemical properties of the substituted aromatic amino acids residues.

**Figure 3:**
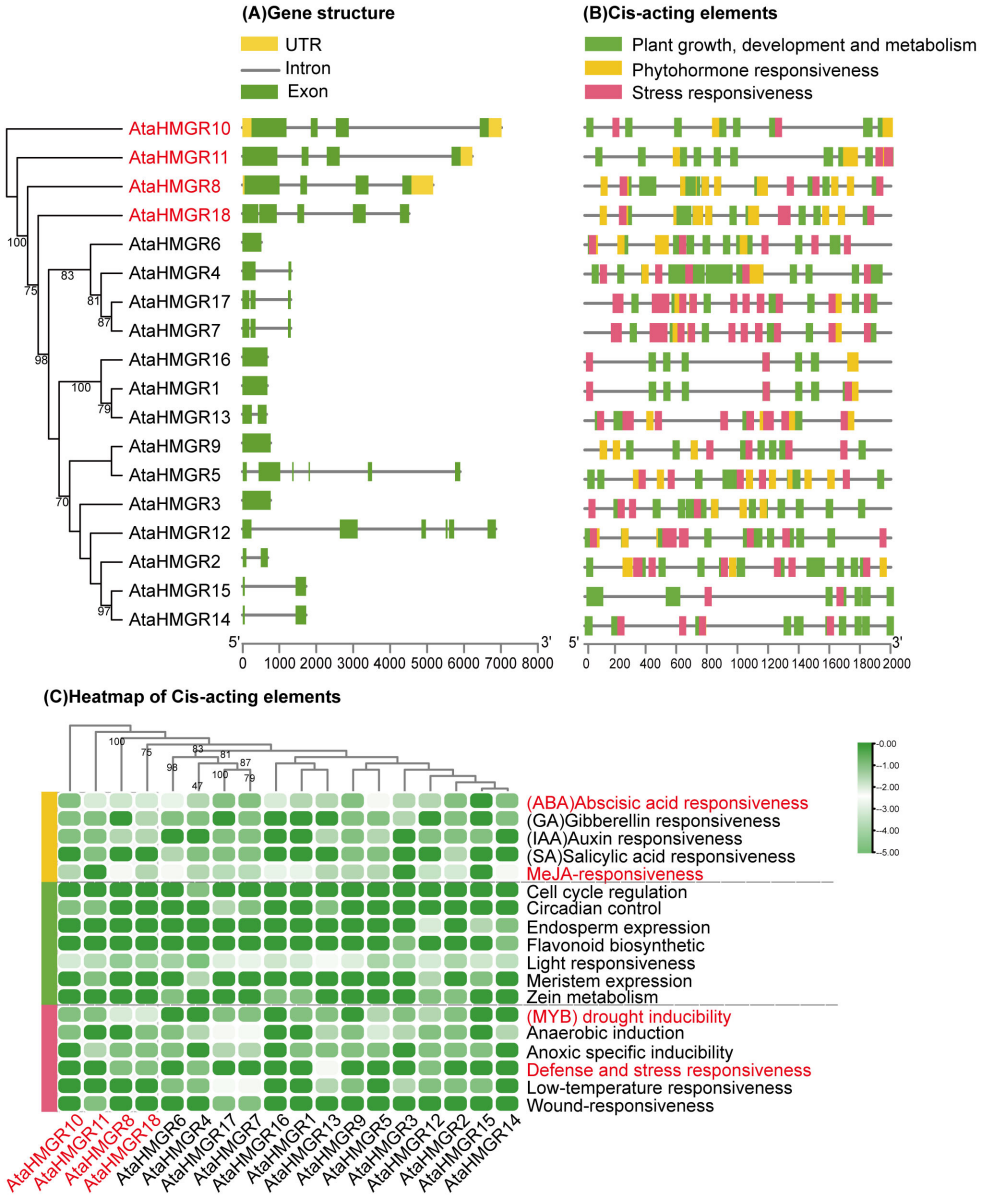
Gene structure and cis-regulatory elements analysis of *AtaHMGRs*. **(A)** Exon-intron structure of 18 *AtaHMGRs*. Green boxes indicate exons, grey lines indicate introns, and yellow boxes indicate UTRs; **(B)** Cis-acting element distribution patterns on 2 kb upstream promoter regions of *AtaHMGRs*; **(C)** The relative cis-element abundance heatmap of *AtaHMGR* gene family, each cell in the grid corresponds to the presence and quantitative abundance of specific cis-elements associated with plant growth, phytohormone, and stress responsiveness, scaled from 0 (no abundance) to 1 (maximum abundance) using TBtools software. Row scaling was applied with a zero-to-one scale method to normalize the data across all genes.

*AtaHMGR* family members clustering on identical phylogenetic branches typically share motif compositions, suggesting functional conservation. The number of motifs across the *A. taliensis* HMGRs ranges from 2 to 12. Notably, most *AtaHMGR* proteins cover over 7 motifs, implying sophisticated architecture for fully functional enzymatic activities. The four genes *AtaHMGR8*, *AtaHMGR10*, *AtaHMGR11*, and *AtaHMGR18* are distinguished by their protein structures with 12 specific motifs, which may form the core functional elements for maintained HMGR activities. The HMGRs (*AtaHMGR8/10/11/18*) structure includes motifs 1 through 5, may be crucial for classical *HMGR* enzymatic functions and other 14 genes without coding sequences of these core motifs of NAD(P)H binding indicates pseudogenes or nova functionalized coding HMGR genes due to adaptation (**Figures 2**, **3**).

In summary, the conserved motifs in the *AtaHMGR* gene family serve as key structural indicators, reflecting the evolutionary lineage and metabolic specialization within *A. taliensis*. The variation in motif presence across the gene family highlights a diverse range of enzymatic functionalities, possibly correlating with adaptation to specific physiological factors or environmental niches.

### 3.5 Gene structure and promoter cis-regulatory element analysis of *AtaHMGRs*

Analysis of *HMGR* gene structure for exon/intron organization revealed that the number of introns per gene varied from 1 to 6, with 44.44% having three exons (**Figure 3A**). Generally, family members of the same group share similar exon/intron patterns, suggesting that phylogenetic relationships within gene family are highly conserved. The results showed that the *AtaHMGR1*, *AtaHMGR3*, *AtaHMGR6*, *AtaHMGR9* and *AtaHMGR16* are non-intron genes, contrasting with *AtaHMGR5* and *AtaHMGR12* having a higher number of exons and introns (6 exons and 5 introns respectively), suggesting these genes may represent pseudogenes or nova functionalized gene forms within this family due to DNA variation leading truncated coding without NAD(P)H binding domain(s) (**Figures 2**, **3**). The genes with intermediate exon-intron structures, like *AtaHMGR8*, *AtaHMGR10*, *AtaHMGR11*, and *AtaHMGR18*, with 4-5 exons and 3-4 introns, might represent diversification but functional HMGRs due to evolutionary adaptations.

Cis-regulatory elements (CREs) play a pivotal role in the precise spatial and temporal regulation of gene expression. Stress-related CREs equipped plants with the abilities to swiftly adapt their physiological and metabolic status in the face of environmental stimulations, including drought, cold, salinity and extreme temperature fluctuations. Eighteen types of cis-elements were predicted from a set of 497 CREs, with phytohormone response, plant growth and development, and stress response being dominant in the promoters of *AtaHMGR* (**Supplementary Table S6**; **Figure 3**). The representative CREs are illustrated in **Figure 3B** and the results showed that *AtaHMGR4* possessed the highest number of CREs, totaling 41 cis-elements, while *AtaHMGR15* had the lowest ones (only 16 elements). These predicted CREs were categorized into three main groups (**Figures 3B, C**): (i) The first group pertains to phytohormone (166 CREs), including abscisic acid (ABA) (49, 9.86%), gibberellins (GAs) (13, 2.62%), auxin (16, 3.22%),salicylic acid (SA) (10, 2.01%), and MeJA-responsiveness (78, 15.69%). Predominantly, *AtaHMGRs* show significant responses to ABA and MeJA consistent with its stress tolerance roles for changes of terpenoids biosynthesis (ii) The second group is associated with plant growth, development, and metabolism (241 CREs), including light-responsive elements (215, 43.26%), circadian control (6, 1.21%), meristem expression (6, 1.21%), endosperm expression (6, 1.21%), zein (seed storage protein) metabolism (5, 1.01%), flavonoid biosynthesis (2, 0.4%), and cell cycle regulation (1, 0.2%). (iii)The third group focuses on stress responsiveness (90 CREs), with categories such as anaerobic induction (26, 5.23%), low-temperature responsiveness (19, 3.82%), MYB related drought inducibility (18, 3.62%), anoxic specific inducibility (16, 3.22%), defense and stress responsiveness (8, 1.61%), and wound responsiveness (3, 0.6%). The CREs for anaerobic induction are notably prevalent, followed by low-temperature responsiveness, MYB related drought inducibility, and anoxic-specific inducibility.

### 3.6 Protein structure analysis of *AtaHMGRs*

**Figure 4:**
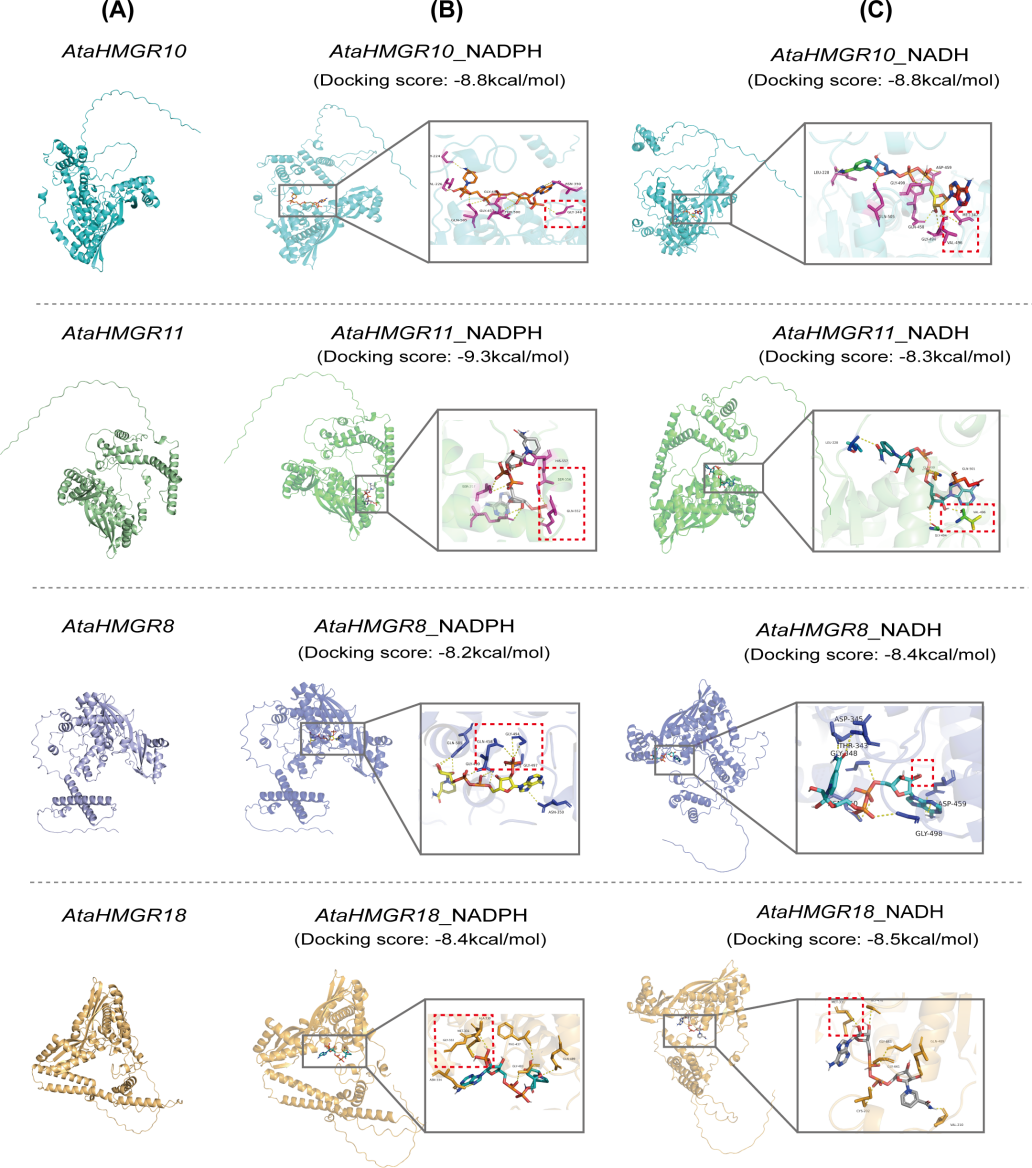
Molecular docking analysis of *AtaHMGR* proteins with substrates NADPH and NADH. **(A)** The 3D structures of four *AtaHMGR* proteins (*AtaHMGR10*, *AtaHMGR11*, *AtaHMGR8*, and *AtaHMGR18*) are shown. **(B)** Molecular docking results of *AtaHMGR/10/11/8/18* with NADPH. **(C)** Molecular docking results of *AtaHMGR10/10/11/8/18* with NADH. The key interaction sites in **(B, C)** are indicated by red dashed boxes.

The analysis of the secondary structures revealed that the 18 *AtaHMGR* proteins had α-helices, extended chains, random coils, and β-turns (**Supplementary Table S7**). The secondary structures of *AtaHMGRs* were categorized into three groups based on their predominant structural components: (i) Group I, including ten proteins (*AtaHMGR1*, *AtaHMGR4*, *AtaHMGR7*, *AtaHMGR8*, *AtaHMGR9*, *AtaHMGR10*, *AtaHMGR11*, *AtaHMGR16*, *AtaHMGR17* and *AtaHMGR18*), were dominated by α-helices compositions, followed by random coils, extended strands, and β-turns, (ii) Group II contained six proteins (*AtaHMGR2*, *AtaHMGR3*, *AtaHMGR5*, *AtaHMGR6*, *AtaHMGR12* and *AtaHMGR13*), with random coils being the most significant feature, followed by the α-helices, extended strands, and β-turns, (iii) Group III comprised of two proteins (*AtaHMGR14* and *AtaHMGR15*) which were characterized by extended strands as the predominant element, followed by random coils, β-turns and α-helices. The 3D structures of the related *AtaHMGR* proteins were generated using online modeling described in the method and materials (**Figure 4**; **Supplementary Figure S3**). Each model was assessed for its quality and reliability based on parameters such as GMQE scores and sequence identity percentages, ensuring the accuracy of predictions. The structural analysis revealed *AtaHMGR8*, *AtaHMGR10*, *AtaHMGR11*, and *AtaHMGR18*, which were grouped together based on phylogenetic analyses (**Figure 2**), showed similar folding patterns and 3D structures likely reflecting their conserved catalytic functions during the evolution. Similarly, *AtaHMGR14* & *AtaHMGR15* as well as *AtaHMGR1*, *AtaHMGR3* & *AtaHMGR16*, exhibited similar 3D structural folding.

Further the analyses of docking of NADH, NAD, NADPH, and NADP to *AtaHMGR8/10/11/18*, containing NAD(P)H binding motifs (**Figure 4**) respectively by AlphaFold3, and the results showed that *AtaHMGR8* and HMGR18 preferred NADH, but *AtaHMGR11* preferred NADPH, while both *AtaHMGR10* did not distinguished NADH and NADPH as reducing agents for catalyzing MVA conversion indicating by its docking scores (the values of reduced free energy after their binding) (**Figure 4**; **Supplementary Table S8**). This comprehensive prediction of 3D structure and NADP(H) docking prediction enhances our understanding of the functional dynamics within the HMGR family of *A. taliensis* and lays the groundwork for future empirical studies aimed at elucidating the biochemical properties and interaction networks and possible evolutional events of these proteins.

### 3.7 Expression characterization of *AtaHMGR* genes in different tissues and MeJA treated seedings

**Figure 5:**
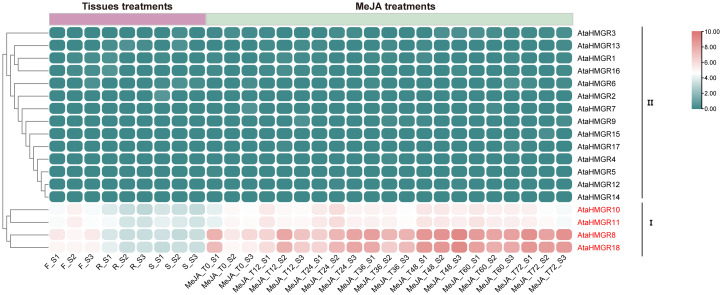
Expression profiles of *AtaHMGR* genes in different tissues and under MeJA treatments. F, flowering twigs; R, roots; S, stems. Time points T0, T12, T24, T36, T48, T60, and T72 indicate the treatment durations of *A. taliensis* plants with MeJA for 0, 12, 24, 36, 48, 60, and 72 hours, respectively.

RNAseq data were utilized to investigate the expression patterns of *AtaHMGR* genes in various organs (i.e., roots, stems, and leaves) of two-year-old *A. taliensis* plants grown in the field, as well as in four-month-old seedlings subjected to MeJA treatment in pots. These expression changes were analyzed to infer the potential biological functions of *AtaHMGR* genes. The detailed expression levels of each gene in different tissues and under MeJA treatments are summarized in **Supplementary Table S9**. The *AtaHMGR* gene family showed varied expressional patterns in response to MeJA, as well as the changed expressions in Rs, Ss and Fs, potentially reflecting the plant’s adaptive mechanisms to environmental stimulations resulting in changes of terpenoids biosynthesis and accumulation. Cluster analysis categorized the tissue-specific expression characteristics of *AtaHMGR* genes into two principal patterns (**Figure 5**). Cluster I included four genes: *AtaHMGR8*, *AtaHMGR10*, *AtaHMGR11*, and *AtaHMGR18.* These genes generally exhibited high and widespread expression during different stages of MeJA treatments, with the highest expression levels observed in flowers (Fs). However, their expression in roots (Rs) and stems (Ss) was significantly lower, suggesting a predominant role of these genes in floral tissues. Among them, *AtaHMGR10* and *AtaHMGR11* showed elevated expression in seedlings at 48 and 60 hours post-MeJA treatments, while *AtaHMGR8* and *AtaHMGR18* displayed slightly higher expression levels compared to *AtaHMGR10* and *AtaHMGR11* under similar conditions. These findings imply that *AtaHMGR8* and *AtaHMGR18* may play more significant roles in MeJA-mediated responses. Cluster II comprised of 14 genes that manifested minimal or no expression under both normal and MeJA inducing conditions, indicating these genes might be pseudogenes or expressed under specific, untested conditions.

### 3.8 Identification of transgenic plants

Bioinformatic analyses revealed an abundance of stress-associated cis-regulatory elements (CREs) in the *AtaHMGR* family members (**Figure 3**), indicative of their potential role in mediating plant stress responses. Distinct expression profiles of *AtaHMGR* genes were observed across various tissues and MeJA treatments; *AtaHMGR8*, *AtaHMGR10*, *AtaHMGR11*, and *AtaHMGR18* showed the highest expression levels, in contrast to the minimal or undetectable expression of the other members (**Figure 5**). Additionally, considering the gene structure, conserved motifs, linear analysis, and protein structure of the *HMGR* genes, *AtaHMGR10* was first chosen as a representative for comprehensive functional analyses. Transgenic *A. thaliana* plants expressing *AtaHMGR10* were generated. We selected two T2-generation of *AtaHMGR10*-overexpression (OE) lines (OE-1 and OE-3) with homozygous *AtaHMGR10* for subsequent analyses. To further investigate *AtaHMGR10* expression in these lines, we used qRT-PCR to examine the expression levels in the transgenic *A. thaliana* plants, and the results showed that *AtaHMGR10* in *AtaHMGR10* transgenic (OEs) lines had higher expression levels (**Supplementary Figure S4**).

### 3.9 Subcellular localization of *AtaHMGR10*

**Figure 6:**
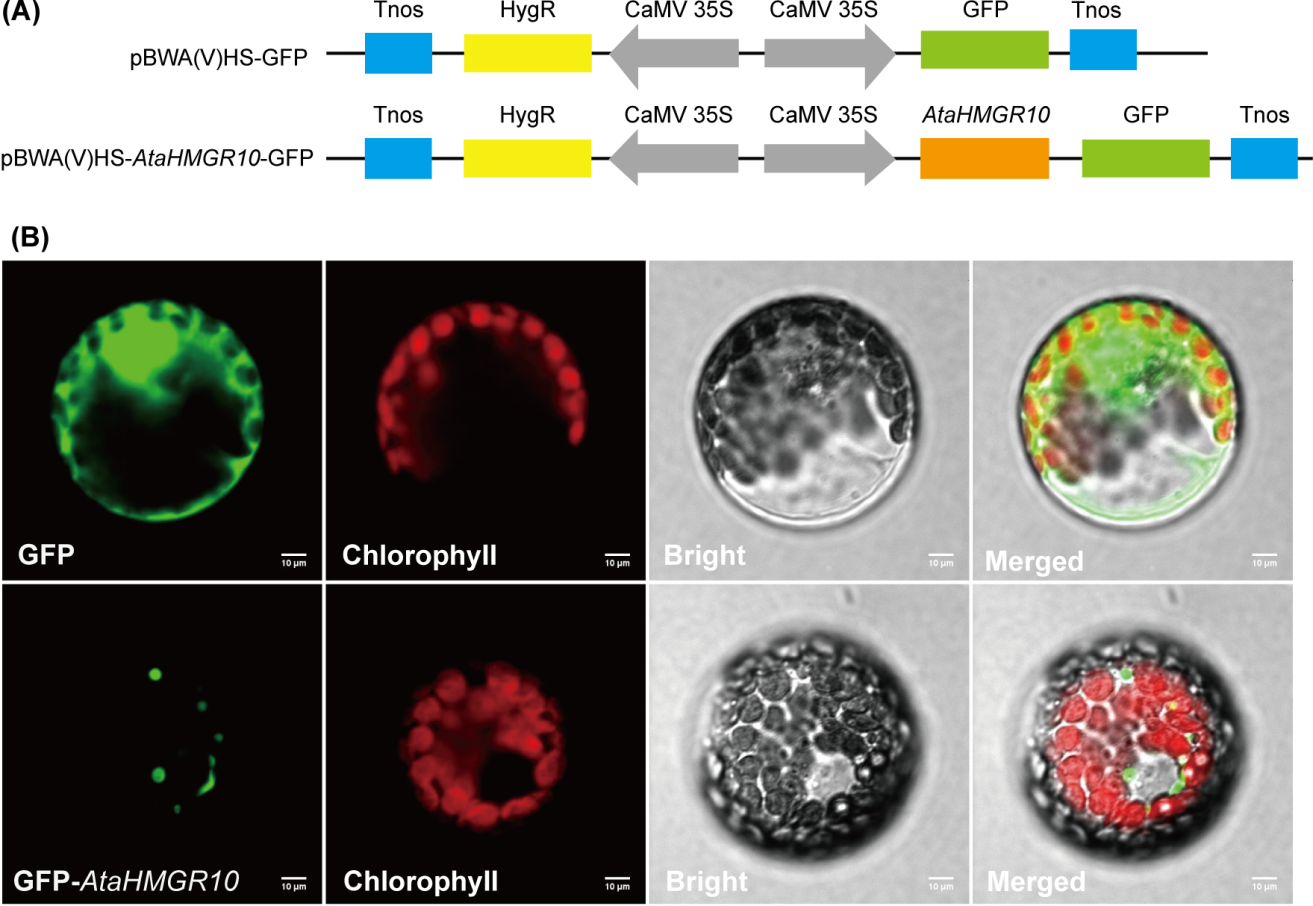
Subcellular localization of *AtaHMGR10* in tobacco protoplasts. **(A)** Vectors for transient expression were created by cloning the full-length coding sequence (CDS) of *AtaHMGR10* into the pBWA(V)HS vector. **(B)** Tobacco leaf protoplasts transfected with either green fluorescent protein (GFP)-*AtaHMGR10* fusion or GFP alone using the polyethylene glycol method. Green fluorescence indicates GFP signals; red indicates chlorophyll autofluorescence; and gray represents the bright field image. Merged images illustrate the overlay of all three channels. Scale bar = 10 μm.

To ascertain the subcellular positioning of *AtaHMGR10*, the CDS were engineered to be fused upstream with the green fluorescent protein (GFP) in the plant expression vector. This recombinant fusion gene was transiently expressed in the protoplasts of tobacco leaves (**Figure 6**). The results showed that *AtaHMGR10* is predominant located in the endomembrane system, indicating a vital role in intracellular signaling and terpenoids precursors biosynthesis [e.g. cholesterol, stress related volatile terpenes as well as phytohormones (e.g. BRs, GAs and, ABA)].

### 3.10 Effect of salt and osmotic stress on seed germination in transgenic *A. thaliana* lines

**Figure 7:**
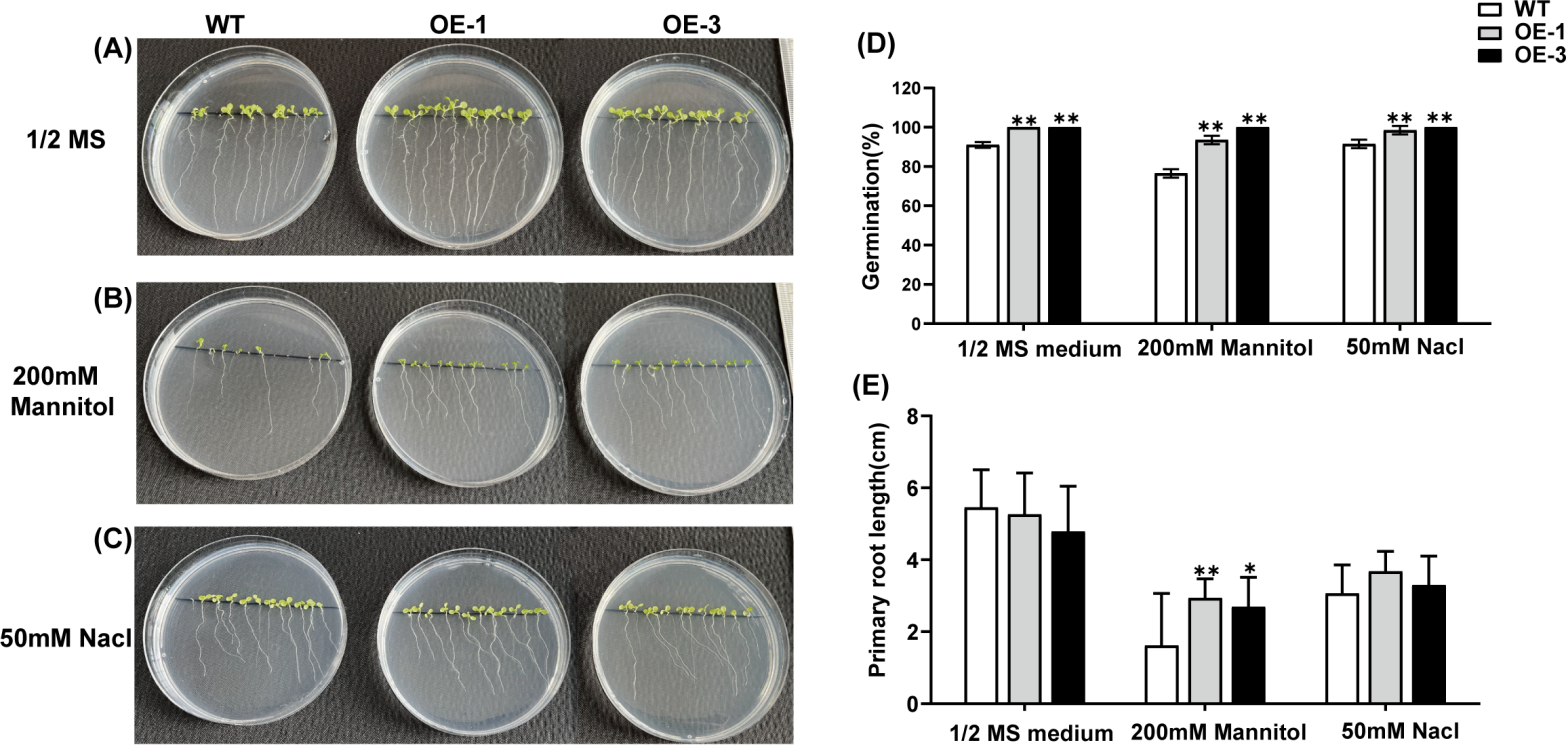
Germination rate and root length comparison of Arabidopsis seedlings under 1/2 MS medium, 200 mM mannitol, and 50 mM NaCl. **(A–C)** Phenotypes of *Arabidopsis* seedlings; **(D)** Germination rate in percentages (%); **(E)** Primary root length comparison of *Arabidopsis* seedlings under the above-mentioned conditions. *p < 0.05, **p < 0.01.

To investigate whether *AtaHMGR10* overexpression enhanced stress tolerance in *A. thaliana* plants, seeds of both WT and transgenic lines with homozygotes *AtaHMGR10* (OE-1, OE-3 of T2 generation) were cultured on 1/2 MS media, with separate sets receiving either 50 mM NaCl or 10% PEG 6000 as simulations of stress treatments (**Figure 7**). Germination rates and phenotypic observations were recorded after 9 days. The germination rates of transgenic lines (OE-1 and OE-3) were significantly higher than WT under 1/2 MS medium with salt and osmotic stress treatments respectively (**Figure 7D**). Under osmotic stress, the primary root length of transgenic plants was longer than that of WT (**Figures 7A, B, E**). This indicates that heterologous expression of *AtaHMGR10* improves the stress tolerance in *Arabidopsis* plants with root growth promotion. There was no significant difference in the primary root length between WT and transgenic plants when grown on 1/2 MS medium under salt stress (50 mM NaCl) conditions (**Figures 7A, C, E**). This suggests that the transgenic plants may not exhibit marked difference from WT on normal growth conditions and low salt stress with 50 mM NaCl simulation.

### 3.11 Overexpression of *AtaHMGR10* confers abiotic stress tolerance with changes in growth and physiological status of transgenic Arabidopsis

**Figure 8:**
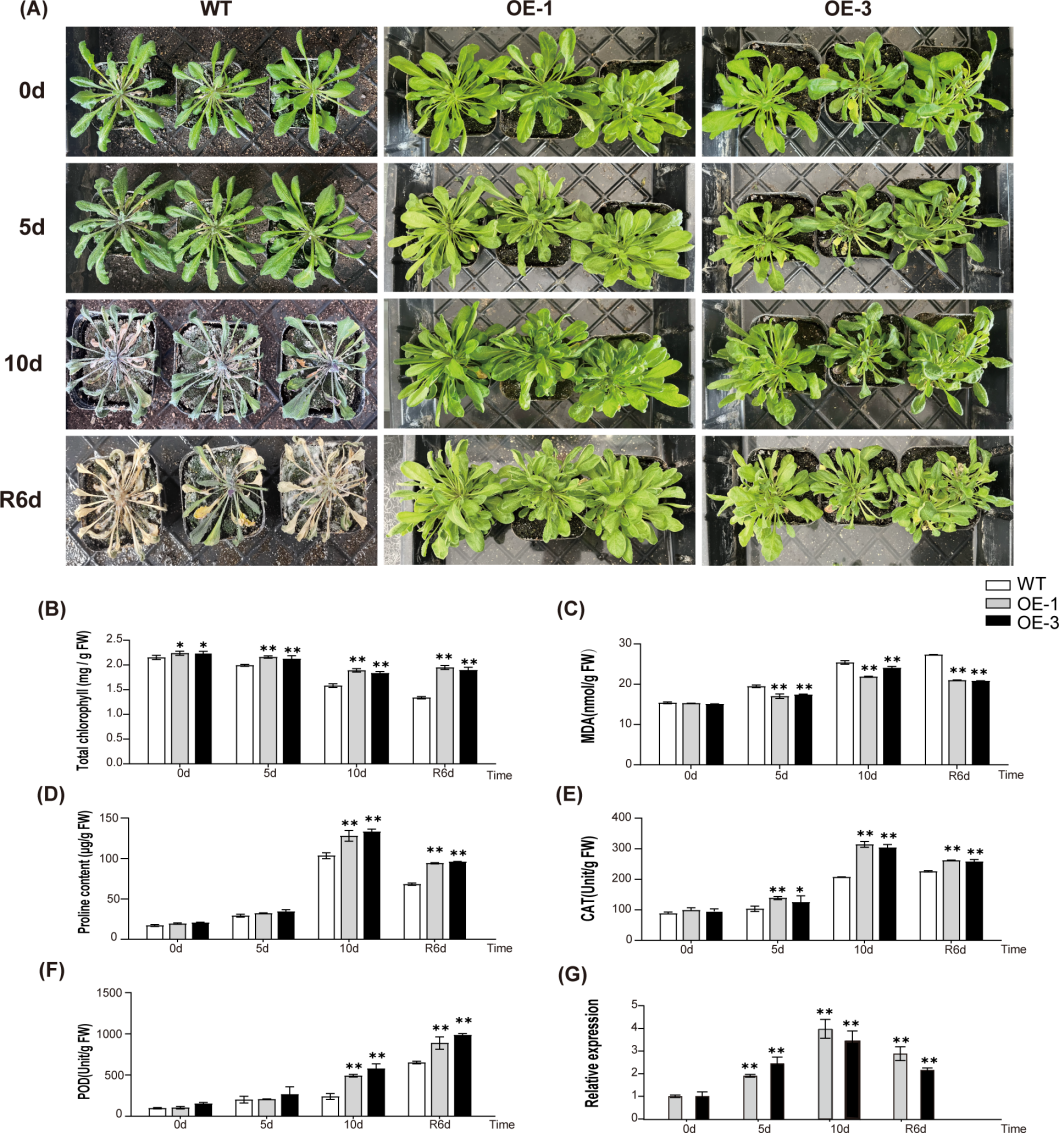
Comparative analysis of drought-induced sensitivity between WT (wild type) and transgenic Arabidopsis. **(A)** Phenotype comparison of WT and transgenic *Arabidopsis*. The labels 0 d, 5 d, and 10 d represent the drought exposure periods, while ‘R6d’ represents rewatering for 6 days following 10 days of drought stress. **(B–F)** Physiological changes in WT and T2 transgenic *A*. *thaliana* (OE-1 and OE-3). **(B)** Changes in chlorophyll contents; **(C)** Changes in MDA contents; **(D)** Changes in proline contents; **(E)** Changes in CAT activities; **(F)** Changes in POD activities; **(G)** Relative expression of *AtaHMGR10*. *p < 0.05, **p < 0.01.

To further explore the impacts of *AtaHMGR10* overexpression on stress tolerance, 6-weeks WT, OE-1 and OE-3 lines were subjected to drought treatment for 0, 5, and 10 days, followed by re-watering for 6 days. Subsequently, the phenotypes of the plants were observed and the collected leaf samples were used to measure the relevant stress related physiological indicators. No observable differences were found in the growth status between WT and transgenic plants under normal growth conditions. However, after 10 days of drought treatment, WT plants exhibited more severe wilting and chlorosis as compared with the OE plants (**Figure 8A**). After 6 days with re-watering, majority of the WT plants died, whereas the transgenic plants gradually recovered.

The stress related physiological indicators and transcriptional responses to abiotic stresses in WT and transgenic *A. thaliana* lines of both OE-1 and OE-3 were illustrated. Under drought stress, a progressive reduction in total chlorophyll contents were observed in both transgenic and WT lines (**Figure 8B**), with transgenic lines displaying higher contents of chlorophylls, particularly at 10 days, hinting *AtaHMGR10*’s involvement in ameliorating chlorophyll degradation compared with the WT. The MDA levels, indicator of lipid peroxidation due to membrane damages, escalated in the WT under drought stress while remaining subdued in lines of both OE-1 and OE-3 (**Figure 8C**), reflecting the heightened oxidative stress resilience in OE-1 and OE-3 by *AtaHMGR10* overexpression. Proline, a stress biomarker, surges in all plants under stress treatments, with OE lines showing a significant elevation (**Figure 8D**), suggesting superior osmotic stress adaptation of OE lines. Antioxidant enzymes activities of both catalases (CATs) and peroxidases (PODs) for removing ROSs damages of membranes were determined and resulted in initial peak activities before declining (**Figures 8E, F**) with the transgenic lines experiencing a more gradual increase than the WTs, help to understand the *AtaHMGR10*’s putative protective role against oxidative stressful injuries.

**Figure 9:**
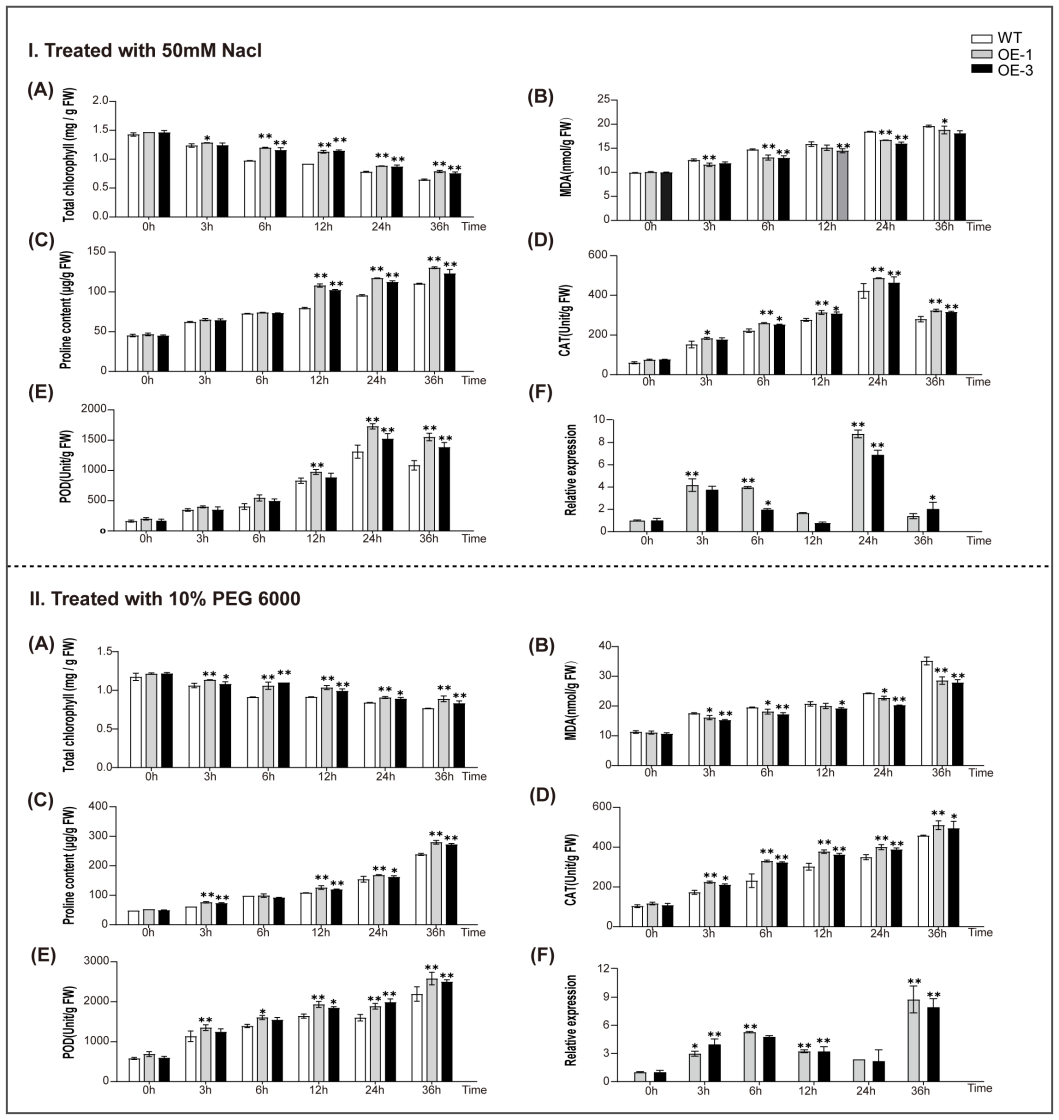
Physiological changes in WT and T2 transgenic *A. thaliana* (OE-1 and OE-3) under **(I)** 50 mM NaCl, and **(II)** 10% PEG 6000 treatments. (IA, IIA) chlorophyll contents; (IB, IIB) MDA contents; (IC, IIC) proline contents; (ID, IID) CAT activities; (IE, IIE) POD activities. (IF, IIF) Relative expression of *AtaHMGR10*. *p < 0.05, **p < 0.01.

Further analyses results showed that during salt stress, chlorophyll concentrations (**Figure 9I**-A) underwent a modest reduction, however, but increased at 24 and 36 hours, especially in WT compared with transgenic lines. The MDA concentrations increased across all samples (**Figure 9I**-B), and the increase were tempered in transgenic lines. Elevated proline levels of transgenic lines (**Figure 9I**-C) suggest a more favorable osmotic equilibrium by *AtaHMGR10* expression. CATs and PODs activities showed an initial upsurge followed by a decrease (**Figures 9I**-D, E), with a less decline in transgenic lines, implying an *AtaHMGR10*-induced antioxidant reactions resistance may be due to terpenoids biosynthesis by *AtaHMGR10* overexpression. Upon exposure to PEG 6000-induced osmotic stress, all plant lines exhibit a slight initial decrease in chlorophyll contents (**Figure 9II**-A), which intensifies over time. The chlorophyll content in transgenic lines declined more slowly compared to the WT. The MDA levels increased (**Figure 9II**-B), yet, the ascent is more tempered in OE-1 and OE-3, hinting at enhanced membrane stability through *AtaHMGR10* overexpression. Transgenic lines accrued significantly more proline contents (**Figure 9II**-C), indicating that *AtaHMGR10* may facilitate augmented osmotic adaptation in transgenic lines manifesting in increased CATs and PODs enzymatic activities (**Figure 9II**-D, E), possibly reflecting an enhanced antioxidant defense.

In summary, under drought, salinity, and osmotic stress, *A. thaliana* OE lines, OE-1 and OE-3, demonstrated increased tolerance compared to the WT. This tolerance was evident from the OE lines having more stable chlorophyll levels during stress stimulations, indicating the transgenes protective effect on photosynthetic apparatus. MDA assays indicated lower lipid peroxidation in the transgenic lines under all stress conditions, highlighting *AtaHMGR10*’s role in enhancing membrane stability and antioxidant protection. Elevated activities of antioxidant enzymatic activities of both PODs and CATs in the transgenic lines further supported a bolstered detoxification mechanism against ROSs-induced damages. Significantly higher proline accumulation in OE-1 and OE-3 suggests *AtaHMGR10*’s involvement in osmotic balance for cellular membrane protection.

## 4 Discussion

The HMGR enzyme, play key roles in MVA pathway for IPP and DMAPP accumulation, generally have key structural features, including two transmembrane helices, two HMG-CoA binding sites (EMPVGY/FVQIP and TTEGCLVA), and two NAD(P)H binding sites (DAMGMNM and GTVGGGT), as discovered by earlier research (Campos and Boronat, 1995; Darabi et al., 2012; Istvan and Deisenhofer, 2000; Leivar et al., 2005). In this study, all *AtaHMGR* proteins were predicted with a single transmembrane helix at the N-terminus, corresponding to motif 5 (ILAIVSLVASLIYLLSFFGIAFVQS), lacking a second transmembrane helix of the functionalized HMGRs. Most *AtaHMGR* proteins contained HMG-CoA binding sites (motifs 3 and 4: EMPVGFVQLP and TTEGCLVA), but lacked NAD(P)H binding sites (motifs 1 and 2: DAMGMNM and GTVGGGT). Notably, motif 3 showed an amino acid variation in its residues, with Y (tyrosine) replaced by F (phenylalanine), diverged from previous research on HMG-CoA binding sites. This divergence reflected broader diversities of plant HMGRs, where amino acid residues at HMG-CoA binding sites may vary substrate selectivity and affinities. *AtaHMGR8*, *AtaHMGR10*, *AtaHMGR11* and *AtaHMGR18* contained motifs 1, 2, 3, 4, and 5, highlighting potential evolutionary conservation within these genes, correlating to their functional capacities. Furthermore, some plant *HMGR* genes in earlier studies also showed sequence variations or even lost the domain entirely. The variations and losses in motifs across *AtaHMGR* genes coding proteins suggest a complex evolutionary pathway leading to pseudogenes or nova functionalized genes. These structural variations are correlate with functional shifts, influencing enzymatic activities of HMGRs, including substrate selectivity, metabolic pathways changes, expression regulation as well as subcellular localization of HMGRs. The divergence observed in motif 3’s amino acid variation aligns with previous research, indicating a broader evolutionary pattern where variations and losses in structural motifs contribute to functional diversity across plant *HMGR* families.

The published studies indicated that the expression levels of *HMGRs* varied in diverse tissues of different plants. In *A. thaliana*, *AtHMGR*1 exhibited ubiquitous expression across all organs, whereas *AtHMGR2* showed specific expression in rapidly growing tissues, implying its role in regulation of growth and development, especially in stress adaptation and developing cues (Enjuto et al., 1994). Similarly, the *HMGR* genes in *Withania somnifera*, *Salvia miltiorrhiza, Lithospermum erythrorhizon*, apple (*Malus pumila*), *Panax notoginseng*, and *Populus* sp., demonstrated distinct expression patterns, primarily concentrated in specific tissues such as flowers, stems, and leaves (Ma et al., 2012; Akhtar et al., 2013; Liu et al., 2016; Wang et al., 2023). Additionally, *HMGR* genes in some crops were found to be expressed in specific tissues, for example, endosperm of seed, suggesting their importance in seed development and storage accumulation (Li et al., 2014). In our study, the highest expression accumulation was observed in flowering twigs (Fs) in *A. taliensis*, with the expression levels of Fs>Rs>Ss consistent with most of the *HMGR* genes in crops exhibiting higher expression levels in flowers and roots.

To explore the linear relationships within the *HMGR* gene family, analysis of gene duplication and selection pressure in *A. taliensis* were conducted. Forty segmental duplicates of gene pairs were identified in this study. Then, the Ka/Ks ratio was calculated to study the selection pressure which affects the segmental duplication of *AtaHMGR* gene pairs. The Ka/Ks ratio of all orthologous gene pairs were less than 1.0 (**Supplementary Table S4**), implying that prior to experienced purification selection pressure, gene duplication events occurred.

To further examine the evolutionary relationships of the *HMGRs*, comparative syntenic patterns were generated from 6 different plant species (4 monocotyledonous, 1 dicotyledonous and a base species of angiosperm). The results indicated that there were more syntenic *HMGR* gene pairs between *A. taliensis* and saponins producing monocot species (**Supplementary Figure S2**; **Supplementary Table S4**). Among the 18 *HMGR* genes in *A. taliensis*, only 3 genes (*AtaHMGR8*, *AtaHMGR10*, *and AtaHMGR11*) exhibited syntenic relationships with genes in all other tested species. The conserved syntenic relationships between these 3 genes and their counterparts in other species imply a shared co-evolutionary history and functional conservation. Conversely, the lack of syntenic relationships for the majority of *HMGR* genes in *A. taliensis* suggests evolutionary divergence, potentially resulting from species-specific adaptations or genetic innovations which may be due to the species of *Asparagus* genus radiating from the cradle center of Africa crossing the Sahara region thus adapting to aridification tolerance (Bentz et al., 2024b). Overall, this analysis provides insights into the evolutionary dynamics and functional diversification of the *HMGR* gene family across species of angiosperm especially the genus *Asparagus*.

The promoters of the *AtaHMGRs* contained abundant cis-acting elements responsive to light, hormones, and abiotic stresses (**Figure 3**). It is demonstrated that *HMGR* members expression are related to the plant abiotic stress responses (Wei et al., 2020; Ma et al., 2023; Zhang et al., 2020). Therefore, we speculate that the *AtaHMGR* genes may be associated with tolerance to abiotic stresses.

Comprehensive expression analysis of the *A. taliensis HMGR* gene family led to the identification of four *AtaHMGR* genes (*AtaHMGR8*, *AtaHMGR10*, *AtaHMGR11*, *AtaHMGR18*) that were associated with plant stress resistance due to the induced expression of MeJA, particularly, *AtaHMGR10* was chosen as the first candidate for functional analyses. The subcellular localization of the *AtaHMGR10*, predominantly within the endomembrane system, suggested a vital role in intracellular signaling and transport processes, essential under stress conditions for terpenoids biosynthesis. Further investigations were conducted to assess its effects on plant stress tolerance through overexpression experiment of *AtaHMGR10 in A. thaliana* with improvements in the stress tolerance of transgenic lines to drought, salt and osmotic stresses (**Figures 8**, **9**) partially supports the above-mentioned proposal.

Under drought and salt stress, plants were induced to accumulate excessive ROSs, causing lipid peroxidation and subsequent MDA production. In response, plant typically establish a complex antioxidant system by enhancing the activities of enzymes such as PODs, and CATs to maintain redox homeostasis and improve the plant’s capability to scavenge ROSs (AbdElgawad et al., 2016; Sánchez-McSweeney et al., 2021; Zhang et al., 2020; Ma et al., 2023; Hu et al., 2022). Under conditions of drought, salt, and osmotic stress, the levels of total chlorophylls, MDA, proline, along with the enzymatic activities of PODs & CATs, were altered in transgenic *A. thaliana* lines overexpressing *AtaHMGR10*. These lines exhibited higher chlorophylls and proline levels, lower MDA concentrations, and increased activities of PODs and CATs compared to the WT (**Figure 9**).

This study provides significant insights into the function of *HMGR* gene family in *A. taliensis*, leading to potential applications in future breeding of crops tolerance improvements as well as medicine terpenoids synthesis in microbial host by genetic modification.

## 5 Conclusions

A total of 18 *AtaHMGR* genes were identified from *A. taliensis*, and their chromosome localization, phylogenetic tree, linear analysis, gene structure, motif distribution, cis-acting elements, and protein structure were characterized. The function and evolution of plant *HMGR* genes are conserved, which allows *HMGR* to serve as a marker for assessing phylogenetic relationships among species. Further functional characterization of *AtaHMGR10*, its coding enzymes with same binding abilities to both NADH and NADPH, in *Arabidopsis* has demonstrated a significant enhancement in abiotic stress tolerance, particularly to drought, osmotic, and salt stresses. This study sheds light on the role of *AtaHMGRs* in enhancing our understanding of their potential functions and providing ways for improving crop abiotic stress resilience by gene modification in the future.

## Data Availability

The original contributions presented in the study are included in the article/**Supplementary Material**. Further inquiries can be directed to the corresponding authors.
