# Supplementary material for: Comprehensive genome-wide analysis of the HMGR gene family of Asparagus taliensis and functional validation of AtaHMGR10 under different abiotic stresses
Source: Front Plant Sci. 2025 Feb 20;16:1455592. doi: 10.3389/fpls.2025.1455592 (PMC11883692; doi:10.3389/fpls.2025.1455592)
Supplement: Supplementary file 1 [file DataSheet1.zip › Data Sheet 1/Data Sheet 1/Figures.docx]

# Additional file 1


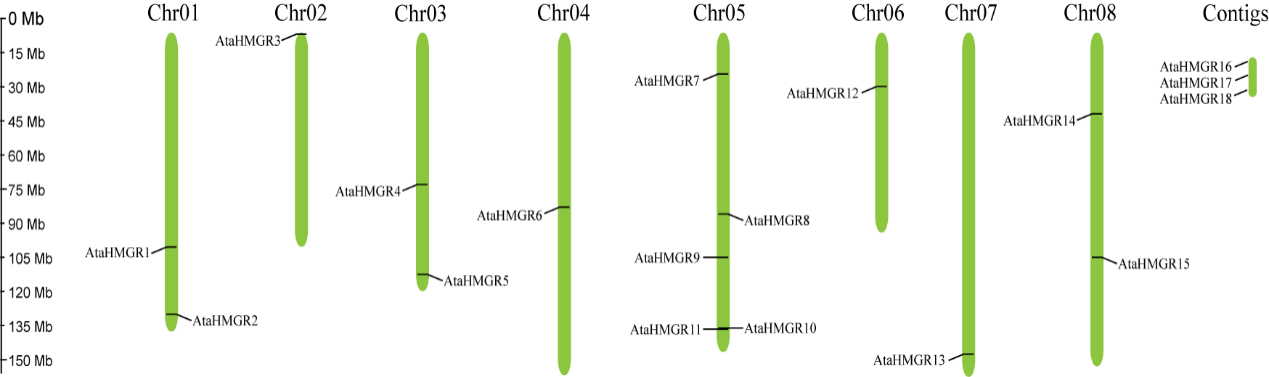


**Figure S1**. Chromosomal distribution of *AtaHMGRs*. The scale on the left is utilized to denote the length of the chromosomes. The green bars represent chromosomes Chr01-Chr08 and contigs. The three genes (*AtaHMGR16*, *AtaHMGR17* and *AtaHMGR18*) are located on contigs.


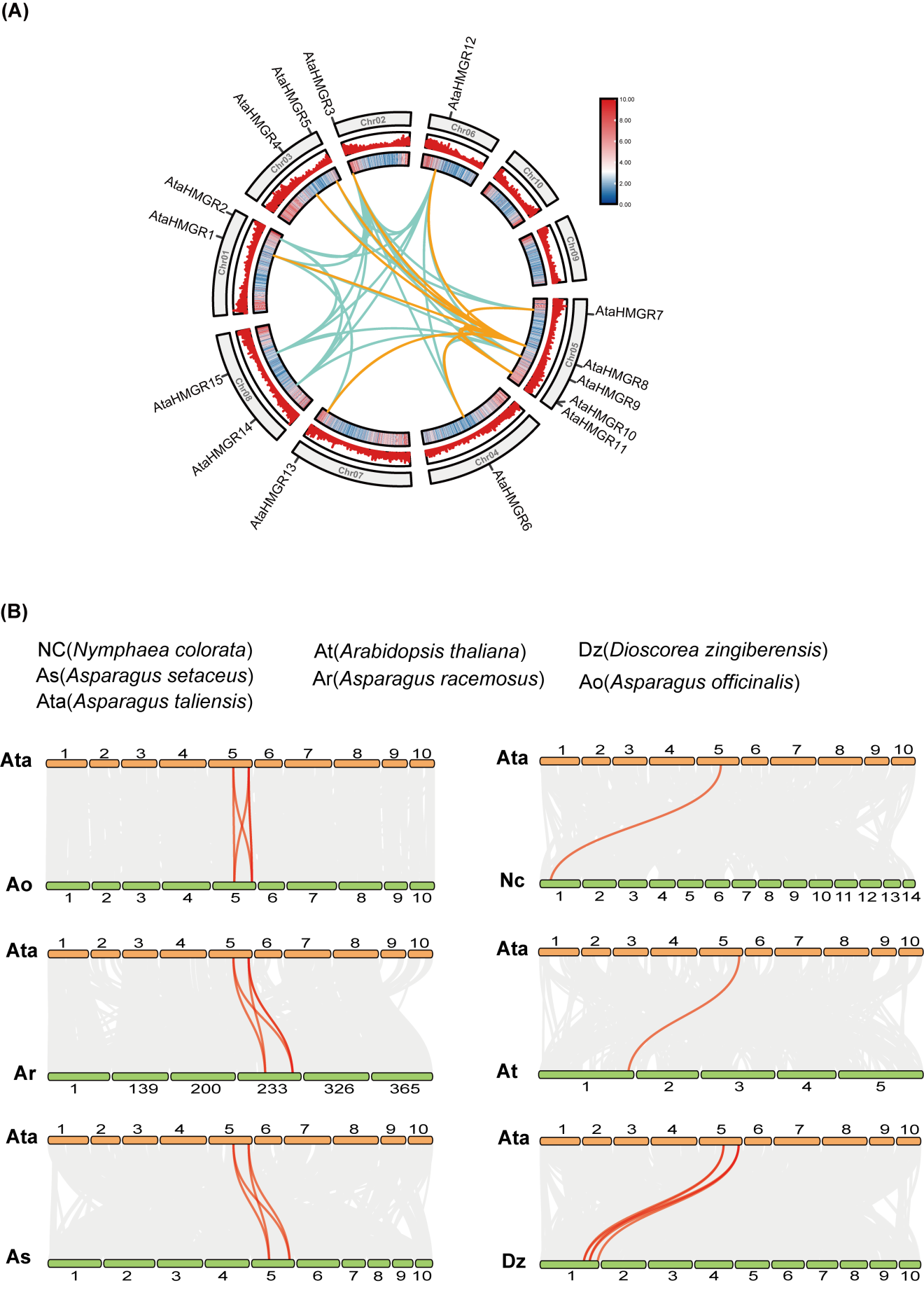


**Figure S2**. Collinearity analysis of *AtaHMGRs* in *A. taliensis*. (A) Distribution and collinearity of *AtaHMGRs* in *A. taliensis* genome. Orange lines represent genome collinearity of *AtaHMGR8*, *AtaHMGR10* and *AtaHMGR11*. The light turquoise lines represent genome collinearity of other 15 *AtaHMGRs*. (B) Synteny analysis of HMGR genes between *A. taliensis* and 6 representative plants.


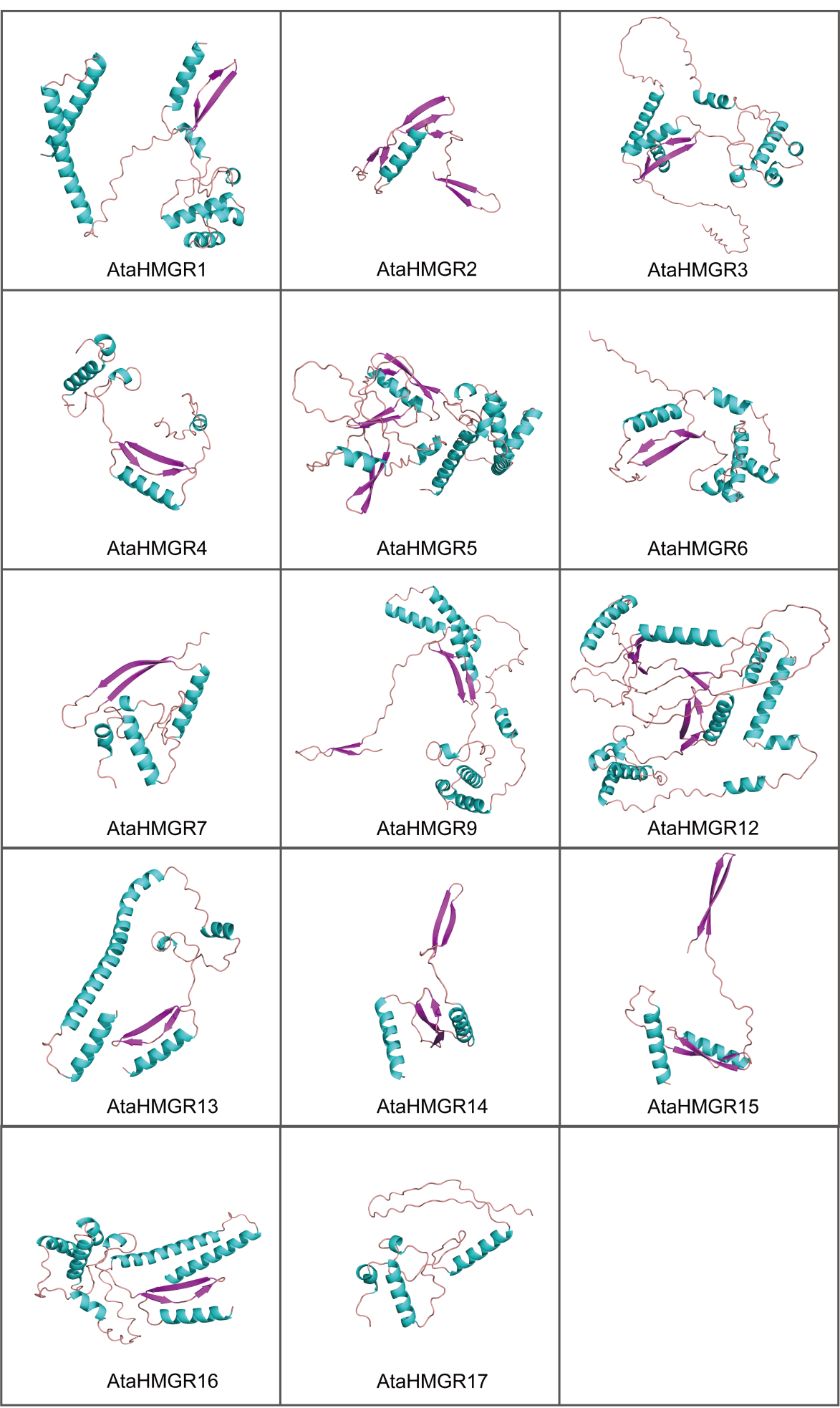


**Figure S3.** 3 D structure analysis of 15 AtaHMGR family members.

**
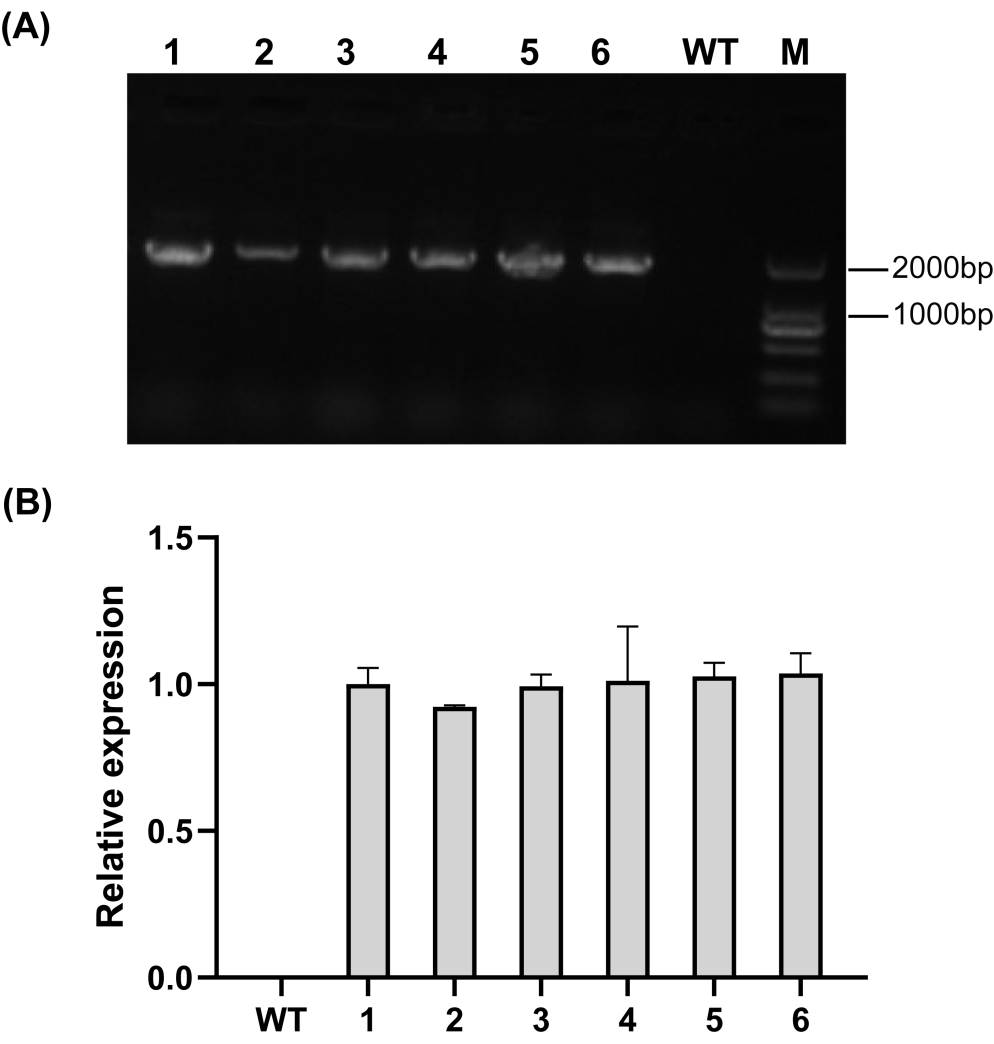
**

**Figure S4.** Identification of transgenic Arabidopsis plants. Analysis of qRT-PCR in WT and OE lines.
